# Supplementary material for: Split-BioID a conditional proteomics approach to monitor the composition of spatiotemporally defined protein complexes
Source: Nat Commun. 2017 Jun 6;8:15690. doi: 10.1038/ncomms15690 (PMC5467174; doi:10.1038/ncomms15690)
Supplement: Supplementary Information — Supplementary Figures, Supplementary Tables and Supplementary Method [file ncomms15690-s1.pdf]

SUPPLEMENTARY FIGURES

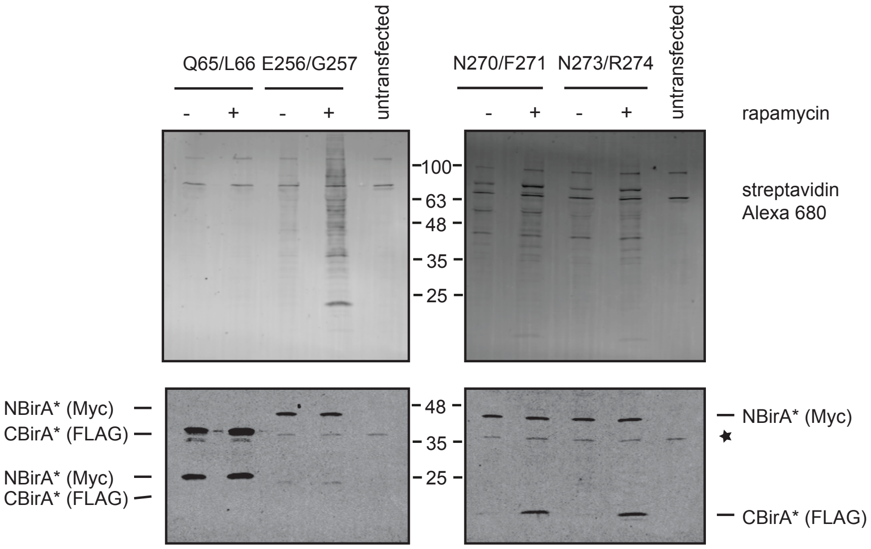

Supplementary Figure 1: Different split sites tested for suitability for a PCA.

Lysates of cells transiently transfected with fusions of NBirA* and CBirA* fragments with FKBP and FRB respectively and incubated with or without rapamycin were analyzed by Western blot for interaction-induced biotinylation. Expression levels of the fusion proteins were detected with the anti-Myc and -FLAG antibodies. The band marked with a star is a non-specific signal. Split sites, as depicted in Fig. 1a, are indicated. Splitting BirA* between E256 and G257 yields two inactive fragments that can reassemble an active enzyme upon interaction of the fusion partners.

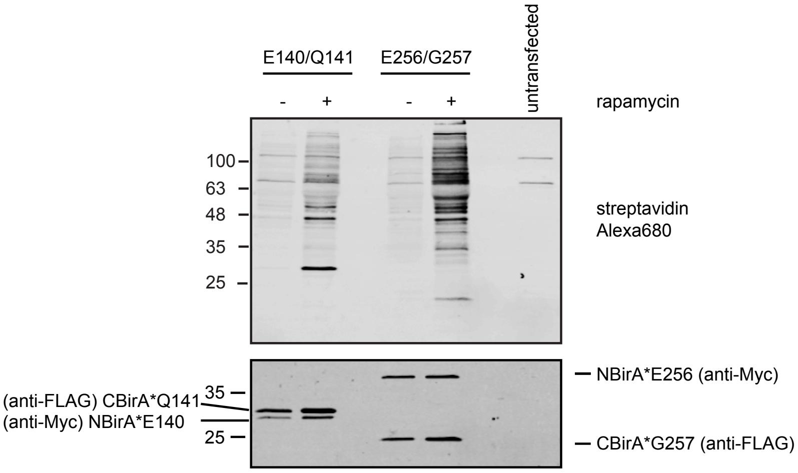

Supplementary Figure 2: Comparison of E140/Q141 and E256/G257 splitBirA* using FRB-FKBP fusions.

Lysates of cells transiently transfected with NBirA*-FKBP and CBirA*-FRB fusions corresponding to the splitting site from De Munter et al. (E140/Q141) or ours (E256/G257) were incubated with or without rapamycin and analyzed by Western blot for interaction-induced biotinylation. Expression levels of the fusion proteins were detected with the anti-Myc and -FLAG antibodies. A stronger biotinylation signal at similar expression levels, indicating a stronger regained activity, is observed in rapamycin-treated cells expressing the E256/G257 construct as compared to the E140/Q141 construct.

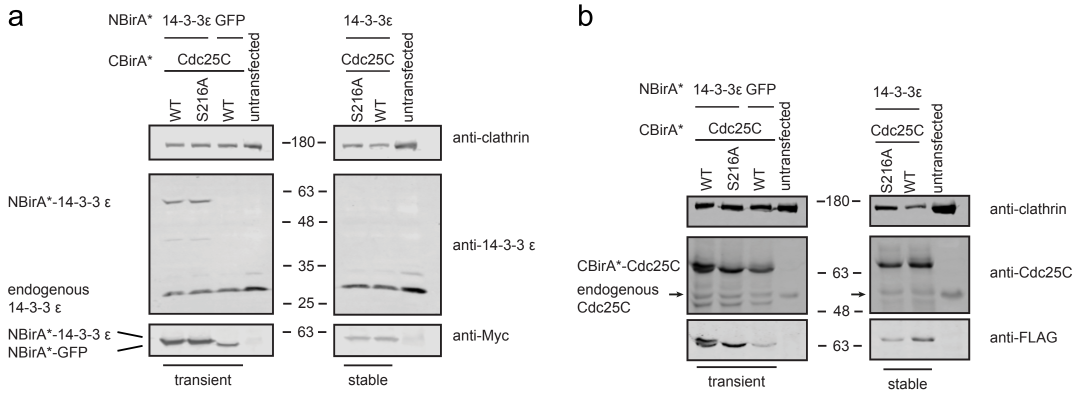

Supplementary Figure 3: Expression levels of BirA* fusion proteins of Cdc25C and 14-3-3ε

Comparison of endogenous and fusion protein expression levels in transiently or stably transfected cells expressing the indicated proteins. (a) Endogenous 14-3-3ε shows higher expression than the corresponding BirA* fusion protein. In stable cell lines NBirA*-14-3-3ε is below the detection limit of the anti 14-3-3ε antibody but expression can be confirmed by Myc detection. (b) CBirA*-Cdc25C protein shows higher expression levels than endogenous Cdc25c in both transient and stable cells. Clathrin detection is used as a loading control.

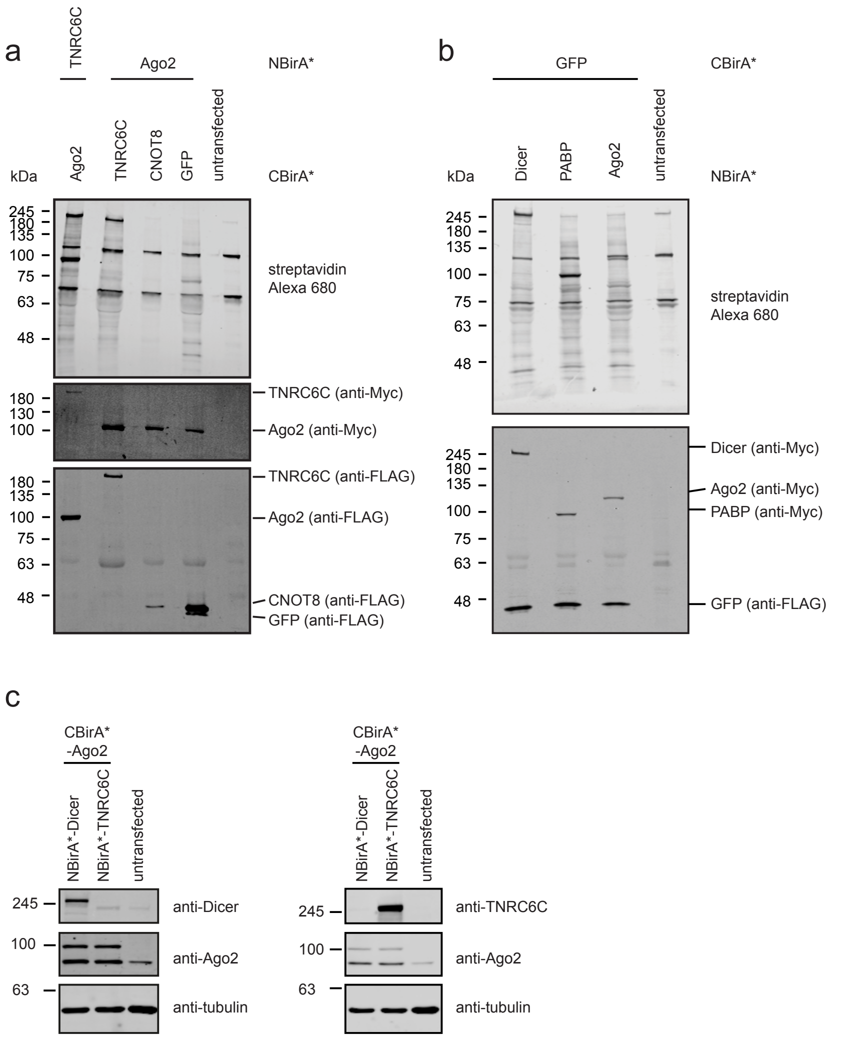

Supplementary Figure 4: Test of various fusion protein orientations for optimal split-BioID of Ago2, Dicer and TNRC6C and comparison of their expression levels with the endogenous proteins

(a) Blot of lysates of cells transiently transfected with the indicated constructs. The NBirA*-TNRC6C/CBirA*-Ago2 combination yielded more biotinylation than the NBirA*-Ago2/CBirA*-TNRC6C counterpart (compare lane 1 and 2) and CBirA*-GFP showed unexpectedly high biotinylation signals (lane 4). (b) Background signals resulting from CBirA*-GFP were further investigated by combining the fusion protein with NBirA* fused to Dicer, Ago2 or PABP. In all cases, strong biotinylation signals could be observed that might be explained by the high expression levels of the CBirA*-GFP fusion. As an NBirA*-GFP fusion yielded background biotinylation levels (Fig. 5b) and the NBirA*-TNRC6C/CBirA*-Ago2 combination resulted in strong biotinylation (a), this orientation was chosen for further experiments. (c) Expression levels of endogenous and fusion proteins in cells transiently transfected with the indicated constructs. Endogenous TNRC6C is not detectable, while endogenous Dicer signals are weaker then the corresponding BirA* fusion protein. Endogenous and CBirA* fusion Ago2 are expressed to the same or lower extent.

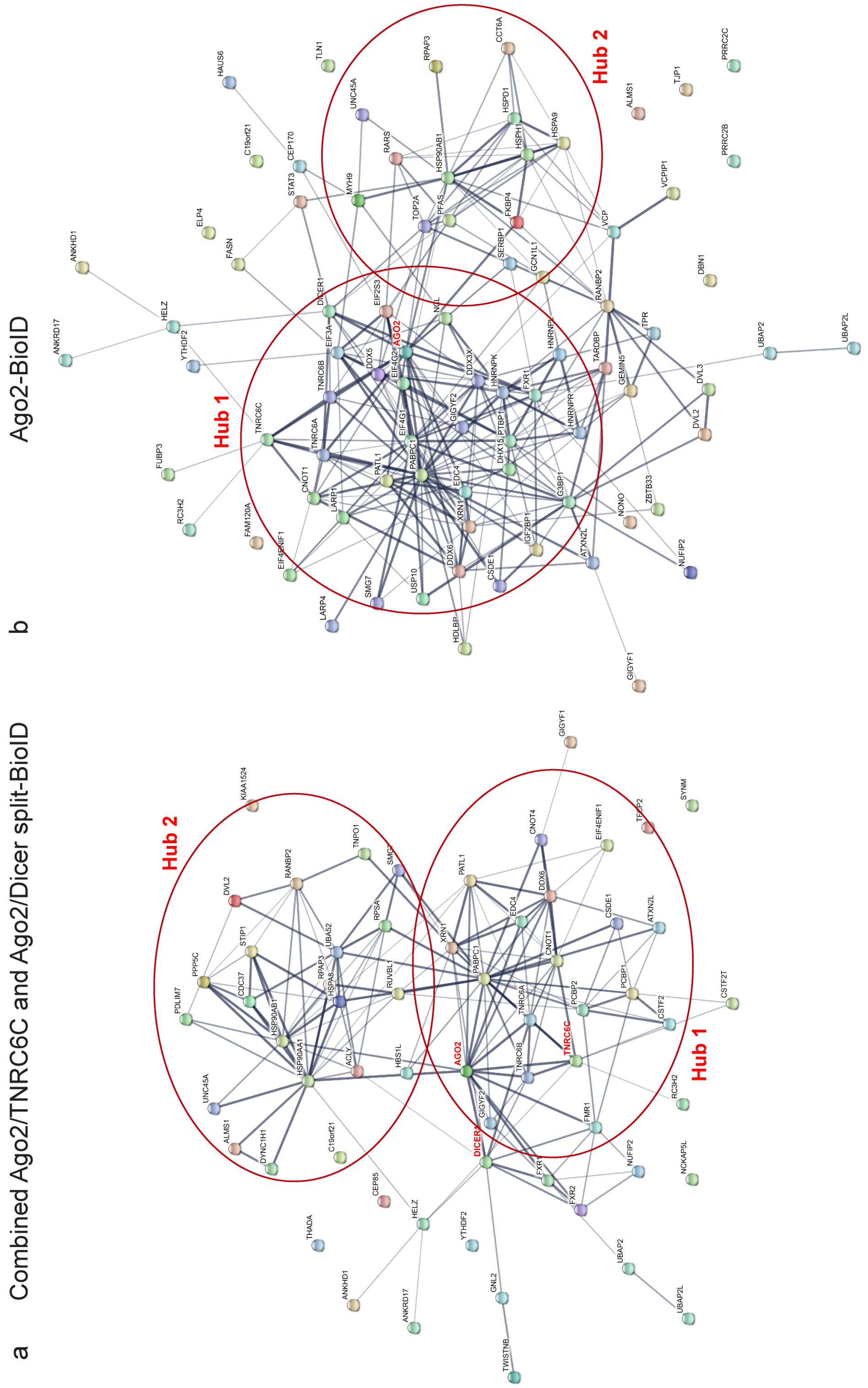

Supplementary Figure 5: String network analysis of the combined Ago2/TNRC6C and Ago2/Dicer split-BioID hits compared to Ago2-BioID. (a) Predicted PPI for the proteins identified in the Ago2/TNRC6C and Ago2/Dicer split-BioID and significantly enriched over the Ago2/GFP split-BioID. Two main interaction hubs centered on PABPC1 (Hub1) and Hsp90 (Hub2) can be observed. (b) Predicted PPI for the proteins identified using standard Ago2-BioID (See Supplementary Data 3). Proteins cluster around the same two hubs as in (a).

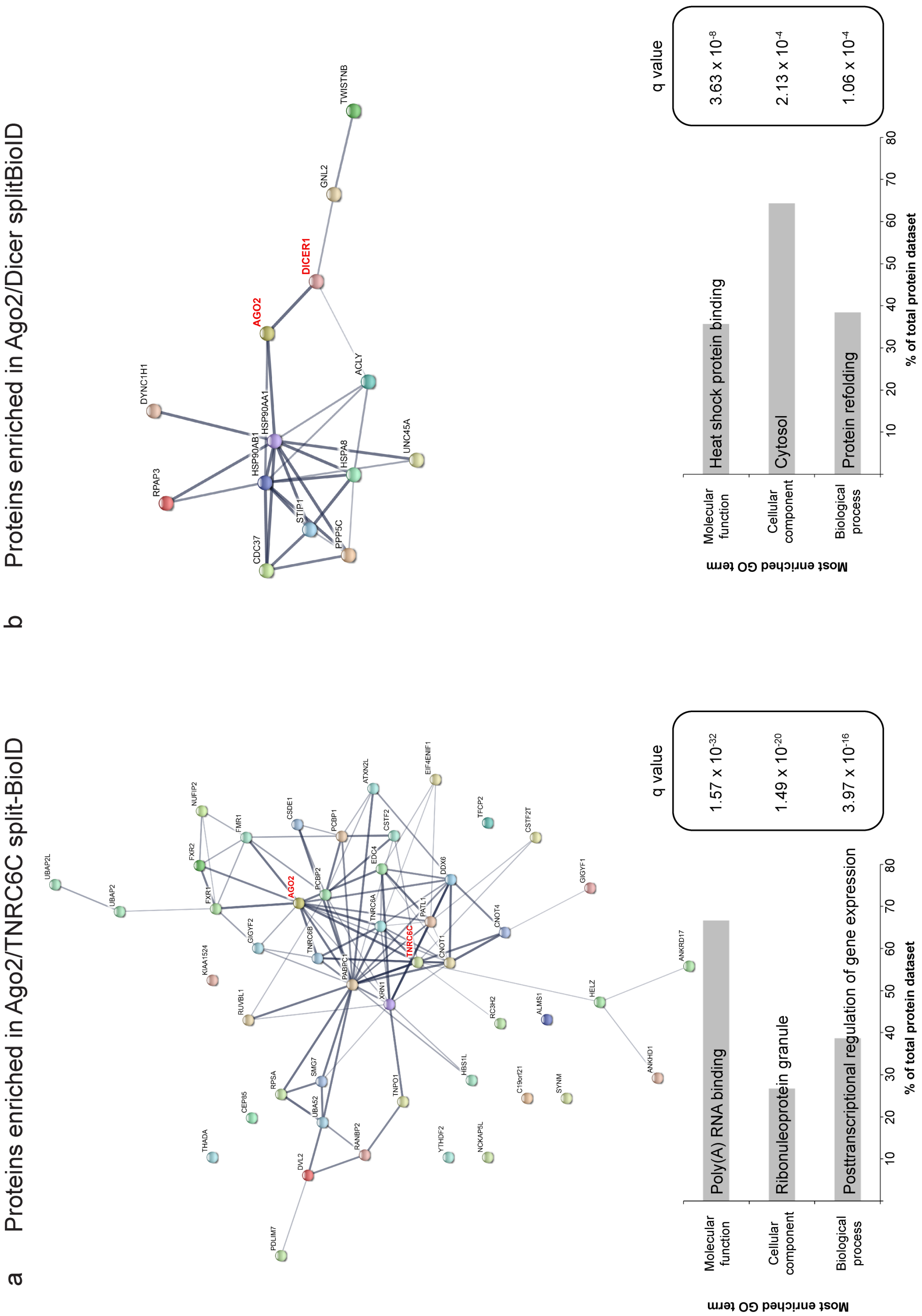

Supplementary Figure 6: String network analysis on the proteins enriched in Ago2/TNRC6C or Ago2/Dicer split-BioID.

(a) Up, predicted PPI for the proteins enriched in the Ago2/TNRC6C split-BioID (see Supplementary Data 2). Down, the corresponding most enriched GO terms are indicated. (b) same as (a) for the proteins enriched in the Ago2/Dicer split-BioID.

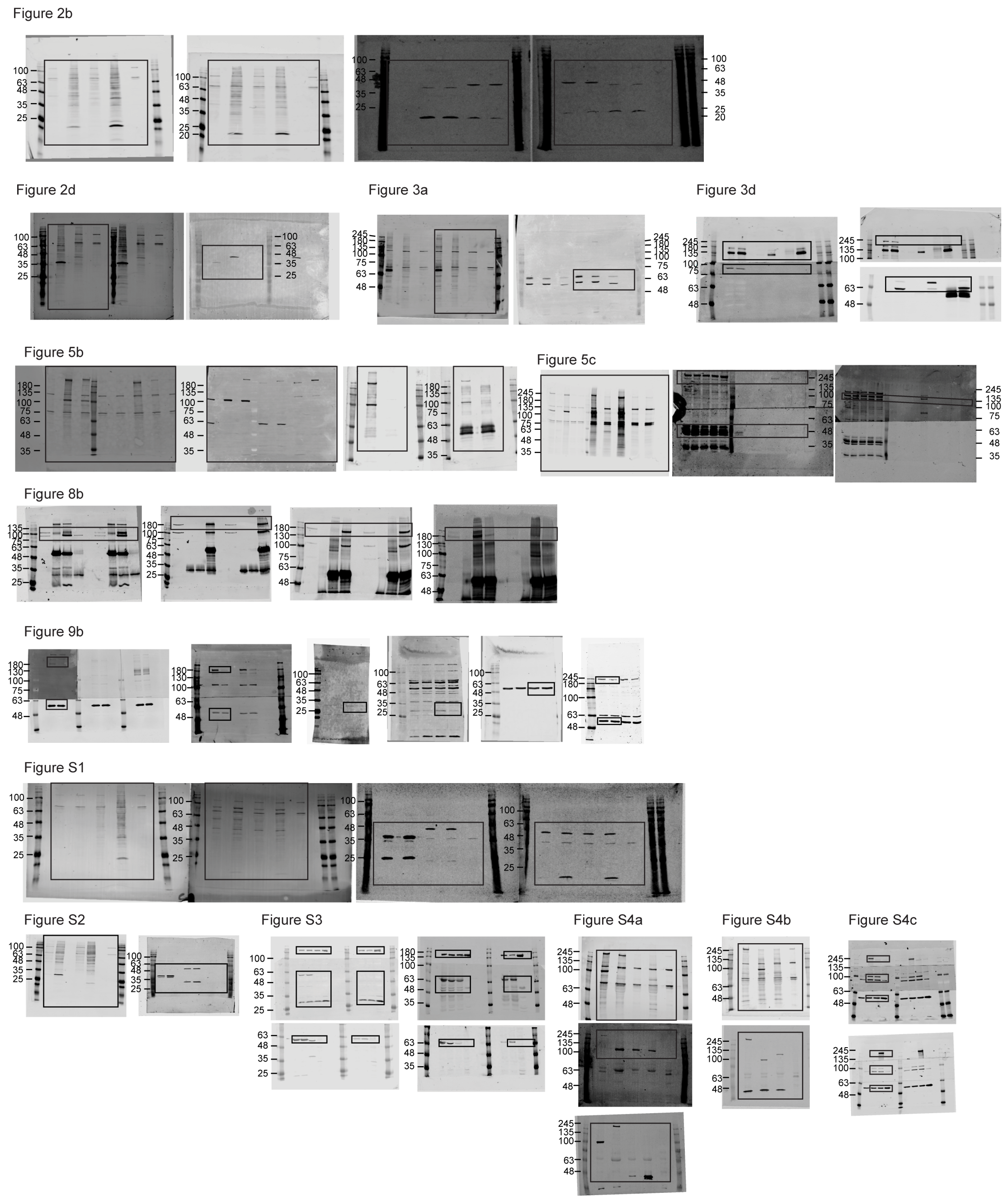

Supplementary Figure 7: Full scans of blots used to prepare the figures. Figures and corresponding cropped areas are indicated.

SUPPLEMENTARY METHODS

Supplementary table 1: Primers and oligos used in this study

Plasmid	Forward primer/oligo	Reverse primer/oligo
pEYFP- Mitotrap<br>(addgene 46942)	CAGCTTGGCCGGCCATGC<br>GAGTGGCCATCCTCTG<br>GTTTAAACATGATCCTCTG<br>GCATGAGAT	ACGAATGCGGCCGCTTAATTA<br>ACTGCTTTGAGATTCGTC<br>ACGAACGATCGCTATTTAATTA<br>ACTGCTTTGAGATTCGTCG
pLXIN-Ap1g1-FKBP<br>(addgene 46946)	CAGCTAGTCGACGGCGCG<br>CCTGGAGTGCAGGTGGAA<br>AC	CATCGTCGGATCCCTACAGTTT<br>TAGAAGCTCCA
	CGTTATATCGATATGGGAG<br>TGCAGGTGGAAAC	TACTCTACGCGTTTCCAGTTTT<br>AGAAGCTCCAC
pSRA-PABP GFP<br>(Stoecklin group)	ATCTTGATCGATATGAACC<br>CCAGTGCCCCC	ATCTTGGGCGCGCCTTAAACA<br>GTTGGAACACCGG
pIRESneo –FLAG/<br>HA Ago2<br>(Meister group)	CGAATAATCGATATGTACT<br>CGGGAGCCGG	CGTTAGACGCGTTCAAGCAAA<br>GTACATGGTGC
pCIneo –NHA-<br>TNRC6C<br>(Chekulaeva group)	CATGGCTACAGGGAGTGC	CTTAATTTAATTAACAGGGACT<br>CCCCGCTGAG
	TCCCTAACGCGTCTAATAA<br>TTTACAGGGACTCCCCGCT<br>GAG	TCCTACGCCTCCCGGGGCATT<br>GAGATGGCTACAGGGAGTGCC
	ATCGGATCCCGTGCCAAAT<br>CTGACAGTG (CED)	AAAAGCGGCCGCTAGCAGGGA<br>CTCCCCGCTGAG (CED)
	GCACCCACGAGGCCAGCT<br>GCAGGGTTAACCAATC<br>(AAGL mutant)	GATTGGTTAACCCTGCAGCTG<br>GCCTCGTGGGTGC (AAGL<br>mutant)
pGST- cdc25c<br>(addgene 10969)	CATGTCCTCTACGGAACTC<br>TTC	CGATTGTTAATTAATGGGCTCA<br>TGTCCTTCACC
pQTEV-YWHAE<br>(addgene 31562)	TCGCATATCGATATGGATG<br>ATCGAGAGGATCTGG	CTGTAGACGCGTCTGATTTTCG<br>TCTTCCACG
pMS2-GFP	ATTCTATCGATATGGTGAG<br>CAAGGGCGAGG	CAGAATTTAATTAACGCGTTGG<br>CTTGTACAGCTCGTC
pSF3-BirA*<br>(unpublished data)	CATAGGGTCGACATGGAAC<br>AAAAACTCATC (BirA* fwd)	AACATGGGATCCGGCGCGCCC<br>AGCTGGATAGGCTCAG (N65)<br>AACATGGGATCCGGCGCGCCG<br>TTGTCCAGCTTCTCCC (N270)<br>AACATGGGATCCGGCGCGCCG<br>TTGATGAAGTTGTCCA (N273)
	CATTCGGGCCGGCCTTAAT<br>TAACCTGAACGCCAAGCAG<br>ATC (C66)<br>CATTCGGGCCGGCCTTAAT<br>TAACTTCATCAACAGACCT<br>GTG (C271)<br>CATTCGGGCCGGCCTTAAT<br>TAACAGACCTGTGAAGCTG<br>AT (C274)	CGGTAAGATCTTTACGGCAGG<br>GTCGCGCGTTCC (BirA* rev)
	TGACAAGGCCGGCCTTCA<br>GGGACCTGCTGCTGCC<br>(C141)	CGTAGGAGATCTCCTCACGCT<br>CGAGCTTCTC
pSF3-NFKBP-<br>CFRB-2 (created in<br>this study)	CGTTATATCGATATGGGAG<br>TGCAGGTGGAAAC	GATGGCAGCAGCGGATCCCTA<br>CTCCAGTCTCCAG (N140)

Plasmid	Forward primer/oligo	Reverse primer/oligo
pCMV-5-HA-CNOT8 (Winkler group)	TATGCCTGCAGCACTTGTGGAG	GCCTGATTAATTAACTGCTGCATGTTGTTGATAA
pCMV- Dicer (Grimm group)	TGAGATGAAAAGCCCTGCTTTG<br>TCCCTAACGCGTCTAATAATAGCTATTGGGAACCTGAG	CGAATATTAATTAAGCTATTGGGAACCTGAGG<br>TCCTACGCCTCCCGGGGCATTGAGATGAAAAGCCCTGCTTTG
pSF3-NBirA*YWHAE-CBirA*cdc25c (created in this study)	CTATATCGCTCCCCGGCGATGCCAGAGAACT	AGTTCTCTGGCATCGCCGGGGAGCGATATAG
pEGFP-GIGYF2 (Freund group)	AAACAATTGATGCAGAAGTGGTATTACAAAGATC (532)<br>AAACAATTGATGCAGGAACGACTGACCAGG (607)	GCGGCCGCTCACTCTTGCTCTAGCTTTGCAGCTTTGG (740)
Linker 1	AACCAGATCTCCTACGCCTCCCGGGGCGGCGGCTCCTCCGGCGGCGGCTTAAT	TAAGCCGCCGCCGGAGGAGCCGCCGCCCCGGGAGGCGTAGGAGATCTGGTTAAT
Linker 2	GCGCCTGGCGGCGGCTCCTCCGGCGGCGGCCAGATCTCCTACGCCTCCCGGGGCGG	CGCGCCGCCCCGGGAGGCGTAGGAGATCTGGCCGCCGCCGGAGGAGCCGCCGCCAGG
FLAG	ATTCATGGACTACAAGGACGACGATGACAAGGCCGG	CCTTGTCATCGTCGTCCTTGTAGTCCATG

Supplementary Table 2: Plasmids generated in this study

Plasmid	Promotor/Resistance	Description
pSF3-BirA*-Ago2	CMV/Amp	BirA*-Ago2
pSF3-BirA*-Rab11a	CMV/Amp	BirA*-Rab11a
pSF3-BirA*-Sec61β	CMV/Amp	BirA*- Sec61β
pSF3-BirA*-RHD4	CMV/Amp	BirA*-RHD4
pSF3-BirA*-GRASP65	CMV/Amp	BirA*-GRASP65
pSF3-BirA*-TGN38	CMV/Amp	BirA*-TGN38
pSF3-BirA*-Lamp1	CMV/Amp	BirA*-Lamp1
pSF3-NBirA*65-CBirA*66	CMV/Amp	NBirA*-FKBP; FRB-CBirA*
pSF3-NBirA*270- CBirA*271	CMV/Amp	NBirA*-FKBP; FRB-CBirA*
pSF3-NBirA*273- CBirA*274	CMV/Amp	NBirA*-FKBP; FRB-CBirA*
pSF3-NFKBP-CFRB-1	CMV/Amp	NBirA*-FKBP; CBirA*-FRB
pSF3-NFKBP-CFRB-2	CMV/Amp	FKBP-NBirA*, CBirA*-FRB
pSF3-NFKBP-CFRB-3	CMV/Amp	NBirA*-FKBP; FRB-CBirA*
pSF3-NFKBP-CFRB-4	CMV/Amp	FKBP- NBirA*; FRB-CBirA*
pSF3-NBirA*E140-CBirA*Q141	CMV/Amp	FKBP-NBirA*, CBirA*-FRB
pSF3-NAgo2-CGFP-1	CMV/Amp	NBirA*-Ago2; CBirA*-GFP
pSF3-NAgo2-CCNOT8-1	CMV/Amp	NBirA*-Ago2; CBirA*-Cnot8
pSF3-NAgo2-CTNRC6C-1	CMV/Amp	NBirA*-Ago2; CBirA*-TNRC6C
pSF3-NAgo2-CDicer-1	CMV/Amp	NBirA*-Ago2; CBirA*-Dicer
pSF3-NGFP-CTNRC6C-1	CMV/Amp	NBirA*-GFP; CBirA*-TNRC6C
pSF3-NGFP-CDicer-1	CMV/Amp	NBirA*-GFP; CBirA*-Dicer
pSF3-NGFP-CAgo2-1	CMV/Amp	NBirA*-GFP; CBirA*-Ago2
pSF3-NPABP-CGFP-1	CMV/Amp	NBirA*-PABP; CBirA*-GFP
pSF3-NDicer-CGFP-1	CMV/Amp	NBirA*-Dicer; CBirA*-GFP
pSF3-NDicer-CAgo2-1	CMV/Amp	NBirA*-Dicer; CBirA*-Ago2
pSF3-NTNRC6C-CAgo2-1	CMV/Amp	NBirA*-TNRC6C; CBirA*-Ago2
pSF3-NYWHAE-Ccdc25C-1	CMV/Amp	NBirA*-YWHAE; CBirA*-cdc25c
pSF3-NYWHAE-Ccdc25C S216A-1	CMV/Amp	NBirA*-YWHAE; CBirA*-cdc25c S216A
pSF3-NGFP-Ccdc25C-1	CMV/Amp	NBirA*-GFP; CBirA*-cdc25c
pMal-c2x-CED His_6	P-lac/Amp	MBP- TNRC6C-CED His_6
pMal-c2x-CED-AAGL-His_6	P-lac/Amp	MBP- TNCR6C-CED-AAGL-His_6
pGEX-6p-GIGYF2-[532-740]	tac/Amp	GST-GIGYF2 (532-740)
pGEX-6p-GIGYF2-[607-740]	tac/Amp	GST_GIGYF2 (607-740)

Supplementary table 3: gBlocks used in this study

gBlock name	Sequence
FLAG- C257Bir – linker 1 – X	CGGCCGGCCTCAATTATGGACTACAAGGACGACGATGA<br>CAAGATTAACGGACTGGCTCCTTACCTGAGCAGATGGGA<br>GAAGCTGGACAACTTCATCAACAGACCTGTGAAGCTGAT<br>CATCGGCGACAAGGAAATCTTCGGCATCTCCAGAGGAAT<br>CGACAAGCAGGGAGCTCTGCTGCTGGAGCAGGACGGAA<br>TCATCAAGCCCTGGATGGGCGGAGAAATCTCCCTGAGAA<br>GCGCAGAGAAGCTCGAGGCGAACCAGATCTCCTACGCC<br>TCCCGGGGCGGCGGCTCCTCCGGCGGCGGCGTTTAAAC<br>ACCCTGCCGTATTAATTAATAACGAGGATCC
X – FLAG – linker 1 – C257Bir	CGGCCGGCCTCAATTGTTTAAACTATGTTAATTAACGACT<br>ACAAGGACGACGATGACAAGCAGATCTCCTACGCCTCCC<br>GGGGCGGCGGCTCCTCCGGCGGCGGCATTAACGGACT<br>GGCTCCTTACCTGAGCAGATGGGAGAAGCTGGACAACTT<br>CATCAACAGACCTGTGAAGCTGATCATCGGCGACAAGGA<br>AATCTTCGGCATCTCCAGAGGAATCGACAAGCAGGGAGC<br>TCTGCTGCTGGAGCAGGACGGAATCATCAAGCCCTGGAT<br>GGGCGGAGAAATCTCCCTGAGAAGCGCAGAGAAGCTCG<br>AGCGGCCGCCACTGTGCTGTCGCGAACGCCGCGCGATG<br>GAACGCGCGACCCTGCCGTAATAACGAGGATCC
X – linker 2 – myc –N256Bir	CTCGAGCTAATCGATTATATGACGCGTGGCGGCGGCTCC<br>TCCGGCGGCGGCCAGATCTCCTACGCCTCCCGGGGCAA<br>ACATGAACAAAAACTCATCTCAGAAGAGGATCTCGACAA<br>GGACAACACCGTGCCCCTGAAGCTGATCGCCCTGCTGG<br>CCAACGGCGAGTTCCACTCTGGCGAGCAGCTGGGAGAG<br>ACCCTGGGAATGAGCAGAGCCGCCATCAACAAGCACAT<br>CCAGACACTGAGAGACTGGGGAGTGGACGTGTTCACCG<br>TGCCTGGCAAGGGCTACAGCCTGCCTGAGCCTATCCAG<br>CTGCTGAACGCCAAGCAGATCCTGGGACAGCTGGATGG<br>CGGAAGCGTGGCCGTGCTGCCTGTGATCGACTCCACCA<br>ATCAGTACCTGCTGGACAGAATCGGAGAGCTGAAGTCCG<br>GCGACGCCTGCATCGCCGAGTACCAGCAGGCTGGCAGA<br>GGAGGCAGAGGACGGAAGTGGTTCAGCCCATTCGGAGC<br>CAACCTGTACCTGTCCATGTTCTGGAGACTGGAGCAGGG<br>ACCTGCTGCTGCCATCGGACTGAGTCTGGTGATCGGAAT<br>CGTGATGGCCGAGGTGCTGAGAAAGCTGGGAGCCGACA<br>AGGTGAGAGTGAAGTGGCCTAATGACCTGTACCTCCAGG<br>ACCGCAAGCTGGCTGGCATCCTGGTGGAGCTGACAGGC<br>AAGACAGGCGATGCCGCTCAGATCGTGATCGGAGCCGG<br>AATCAACATGGCCATGAGAAGAGTGGAGGAGAGCGTGG<br>TGAACCAGGGCTGGATCACCCTGCAGGAGGCTGGCATC<br>AACCTGGACCGGAACACCCTGGCCGCCATGCTGATCAG<br>AGAGCTGAGAGCCGCTCTGGAGCTGTTCGAGCAGGAGA<br>AACATGGATCCTAGGGCGCGCC

gBlock name	Sequence
Myc- N256Bir – linker 2 – X	CTCGAGATGGAACAAAAACTCATCTCAGAAGAGGATCTC<br>GACAAGGACAACACCGTGCCCCTGAAGCTGATCGCCCT<br>GCTGGCCAACGGCGAGTTCCACTCTGGCGAGCAGCTGG<br>GAGAGACCCTGGGAATGAGCAGAGCCGCCATCAACAAG<br>CACATCCAGACACTGAGAGACTGGGGAGTGGACGTGTT<br>CACCGTGCCTGGCAAGGGCTACAGCCTGCCTGAGCCTA<br>TCCAGCTGCTGAACGCCAAGCAGATCCTGGGACAGCTG<br>GATGGCGGAAGCGTGGCCGTGCTGCCTGTGATCGACTC<br>CACCAATCAGTACCTGCTGGACAGAATCGGAGAGCTGAA<br>GTCCGGCGACGCCTGCATCGCCGAGTACCAGCAGGCTG<br>GCAGAGGAGGCAGAGGACGGAAGTGGTTCAGCCCATTC<br>GGAGCCAACCTGTACCTGTCCATGTTCTGGAGACTGGAG<br>CAGGGACCTGCTGCTGCCATCGGACTGAGTCTGGTGAT<br>CGGAATCGTGATGGCCGAGGTGCTGAGAAAGCTGGGAG<br>CCGACAAGGTGAGAGTGAAGTGGCCTAATGACCTGTACC<br>TCCAGGACCGCAAGCTGGCTGGCATCCTGGTGGAGCTG<br>ACAGGCAAGACAGGCGATGCCGCTCAGATCGTGATCGG<br>AGCCGGAATCAACATGGCCATGAGAAGAGTGGAGGAGA<br>GCGTGGTGAACCAGGGCTGGATCACCCTGCAGGAGGCT<br>GGCATCAACCTGGACCGGAACACCCTGGCCGCCATGCT<br>GATCAGAGAGCTGAGAGCCGCTCTGGAGCTGTTCGAGC<br>AGGAGAAACATGGCGGCGGCTCCTCCGGCGGCGGCCA<br>GATCTCCTACGCCTCCCGGGGCATCGATTATTAGACGCG<br>TTAGGGATCCGGCGCGCC

Supplementary table 4: Antibodies used in this study

Antibody	Host	Dilution	Buffer
anti-Myc (DSHB, 9E10-c)	mouse	WB,1:1000;<br>IF, 1:50	1 % BSA/ 0.1% PBS – Tween 20<br>5% BSA, 2% donkey serum, 2% goat serum 0.02% PBS-Triton-X 100
anti-Myc (Proteintech, 66004-1-Ig)	mouse	IP, 2.5 µg	IP wash buffer
anti-FLAG M2 (Sigma, F1804)	mouse	WB, 1:500<br>IP, 5 µg	2% BSA / 0.1% PBS – Tween 20
anti-FLAG (Sigma, F7425)	rabbit	IF, 1:50	5% BSA, 2% donkey serum, 2% goat serum 0.02% PBS-Triton-X 100
anti-CNOT1 (Proteintech, 14276-1-AP)	rabbit	WB, 1:500	5% milk / 0.2% PBS –Tween 20
anti-CNOT7 (Abnova, H00029883-M01)	mouse	WB, 1:500	3% BSA / 0.1% TBS –Tween 20
anti-CNOT8 (LSBio, LS-C99242)	rabbit	WB, 1:500	2% BSA / 0.1% PBS – Tween 20
anti-TRBP (Proteintech, 15753-1-AP)	rabbit	WB, 1:1000	2% BSA / 0.1% PBS – Tween 20
anti-LMO7 (Santa Cruz, sc-376807)	mouse	WB, 1:200	2% BSA / 0.1% PBS – Tween 20
anti-CKAP5 (Santa Cruz, sc-374394)	mouse	WB, 1:200	2% BSA / 0.1% PBS – Tween 20
anti-rabbit IgG IRDye 800CW (LI-COR, 926-32211)	goat	WB, 1:10000	2% BSA / 0.1% PBS – Tween 20
anti-mouse IgG Alexa Fluor 680 (Thermo scientific, A-21057)	goat	WB, 1:10000	2% BSA / 0.1% PBS – Tween 20
Streptavidin protein, DyLight 680 conjugate (Thermo scientific, 21848)	-	WB, 1:150000	2% BSA / 0.1% PBS – Tween 20
anti-Ago2 (Ascenion, 11A9)	rat	WB, 1:3000;<br>IP, 5 µg;<br>IF, 1:500	5% milk / 0.1% PBS –Tween<br>5% BSA, 2% donkey serum, 2% goat serum 0.02% PBS-Triton-X 100
anti-TNRC6A (Bethyl, A302-329A)	rabbit	WB, 1:3000	5% milk / 0.1% PBS –Tween
anti-TNRC6A (Bethyl, A302-330A)	rabbit	IP, 5 µg	
anti-TNRC6C (Bethyl, A303-969A)	rabbit	WB, 1:1000	2% BSA / 0.1% PBS – Tween 20
anti- GIGYF2 (Novusbio, NBP2-12812)	rabbit	WB, 1:3000;<br>IP, 5 µg;<br>IF, 1:500	5% milk / 0.05% PBS –Tween<br>5% BSA, 2% donkey serum, 2% goat serum 0.02% PBS-Triton-X 100
anti-α-tubulin (Sigma, T5168)	mouse	WB, 1:10000	2% BSA / 0.1% PBS – Tween 20
anti-Cdc25c (cell signaling, #4688)	rabbit	WB, 1:1000	5% BSA / 0.1% TBS – Tween 20
anti-14-3-3 ε (cell signaling, #9635)	rabbit	WB, 1:1000	5% BSA / 0.1% TBS – Tween 20

Antibody	Host	Dilution	Buffer
anti-Clathrin (BD, 610499)	mouse	WB, 1:2000	2% BSA / 0.1% PBS – Tween 20
IgG from rabbit serum (Sigma, I5006)	rabbit	IP, 5µg	
anti-mouse Alexa 488 (Jackson ImmunoResearch, 715-545-150)	donkey	IF, 1:1000	5% BSA, 2% donkey serum, 2% goat serum 0.02% PBS-Triton-X 100
anti-rat Alexa 647 (Invitrogen, A11077)	goat	IF, 1:1000	5% BSA, 2% donkey serum, 2% goat serum 0.02% PBS-Triton-X 100
anti-rabbit Alexa 568 (Invitrogen, A10042)	donkey	IF, 1:1000	5% BSA, 2% donkey serum, 2% goat serum 0.02% PBS-Triton-X 100
streptavidin Cy5 (Jackson ImmunoResearch, 016-170-084)	-	IF, 1:200	5% BSA, 2% donkey serum, 2% goat serum 0.02% PBS-Triton-X 100

Supplementary table 5: IF min/max displayed values

**Hela 11ht** (Fig 3)	**Cy5**	**Alexa488/GFP**	**Dapi**	**Alexa568**
untransfected	556/2612	654/889	566/827	1063/3396
CBirA*-Ago2<br>NBirA*-TNRC6C		654/889		
CBirA*-Ago2<br>NBirA*-Dicer		654/1807		
CBirA*-Ago2<br>NBirA*-GFP		654/889		
**Hela 11ht** (Fig 2)	**Cy5**	**Alexa488/GFP**	**Dapi**	**Alexa568**
untransfected	493/710	426/872	494/827	477/578
CBirA*-Cdc25C<br>NBirA*-14-3-3 **ε**	428/982	426/872		
CBirA*-Cdc25C<br>S216A<br>NBirA*-14-3-3 **ε**		494/972		
CBirA*-Cdc25C<br>NBirA*-GFP		462/546		
**Hela 11ht** (Fig 4)	**Alexa647**	**-**	**Dapi**	**Alexa568**
untransfected	479/692		527/1342	468/761
